# *Vrk1* partial Knockdown in Mice Results in Reduced Brain Weight and Mild Motor Dysfunction, and Indicates Neuronal VRK1 Target Pathways

**DOI:** 10.1038/s41598-018-29215-x

**Published:** 2018-07-26

**Authors:** Hadar Vinograd-Byk, Paul Renbaum, Ephrat Levy-Lahad

**Affiliations:** 1 Medical Genetics Institute, Shaare Zedek Medical Center, Jerusalem, 91031 Israel; 2 Hebrew University Medical School, Jerusalem, 91120 Israel

## Abstract

Mutations in *Vaccinia-related kinase 1* (*VRK1*) have emerged as a cause of severe neuronal phenotypes in human, including brain developmental defects and degeneration of spinal motor neurons, leading to Spinal Muscular Atrophy (SMA) or early onset Amyotrophic Lateral Sclerosis (ALS). Vrk1 gene-trap partial Knockout (KO) mice (Vrk1^GT3/GT3^), which express decreased levels of *Vrk1*, are sterile due to impaired gamete production. Here, we examined whether this mouse model also presents neuronal phenotypes. We found a 20–50% reduction in *Vrk1* expression in neuronal tissues of the Vrk1^GT3/GT3^ mice, leading to mild neuronal phenotypes including significant but small reduction in brain mass and motor (rotarod) impairment. Analysis of gene expression in the Vrk1^GT3/GT3^ cortex predicts novel roles for VRK1 in neuronal pathways including neurotrophin signaling, axon guidance and pathways implicated in the pathogenesis of ALS. Together, our studies of the partial KO Vrk1 mice reveal that even moderately reduced levels of Vrk1 expression result in minor neurological impairment and indicate new neuronal pathways likely involving VRK1.

## Introduction

Recessive inherited mutations in the gene encoding *Vaccinia-related kinase 1* (*VRK1*), a serine-threonine kinase involved in cell cycle regulation and the DNA damage response^1–6^, result in a neurological disease that has both developmental and degenerative manifestations. Neurodevelopmental disease associated with VRK1 deficiency manifests as microcephaly of prenatal onset^7–11^, and may also include other brain malformations, e.g. pontocerebellar hypoplasia^7,10^, underdeveloped cerebellar vermis^9^ and/or simplified gyral pattern^8,9^. These phenotypes have been observed with the complete loss of function R358X mutation^7–9^, as well as in homozygotes for the R133C mutation^10^ and in compound heterozygotes for other VRK1 mutations (G226A and R89Q^9^, G135R and L195V^11^). We recently demonstrated that in the nervous system, VRK1 plays an important role in neuronal migration in the developing cortex, through an amyloid β precursor protein (APP) dependent mechanism, and this role is at least partially non-catalytic^8^. In addition, we showed that VRK1 plays kinase-dependent roles in cell cycle progression of cortical neural precursors^8^.

The neurodegenerative disease associated with VRK1 mutations manifests in all patients as progressive spinal motor neuron degeneration of early childhood to early adulthood onset. Affected children suffer from spinal muscular atrophy (SMA)^7–10^ or motor and sensory axonal neuropathy^9,11^. In adults, the disease has been clinically defined as distal SMA^11^ or early-onset amyotrophic lateral sclerosis (ALS)^12^.

A Vrk1 partial knockout mouse model has previously been described^13–16^. The mutant mice (Vrk1^GT3/GT3^) are homozygous for a gene-trap insertion into intron 3 of *Vrk1*, which includes a strong splice acceptor site at its 5′ end, a β−GEO fusion transcript, and a polyA site at the 3′ end. Integration of the trap into the *Vrk1* gene results in a truncated *Vrk1* transcript containing only the first 3 exons of *Vrk1* fused to the gene trap insertion. The resulting truncated protein lacks the kinase domain and the nuclear localization signal, and is therefore inactive. However, due to incomplete aberrant splicing of *Vrk1* to the trap, Vrk1^GT3/GT3^ mice retain some expression of the full length, functional Vrk1 transcript and protein (15%-30% relative to VRK1^+/+^ mice^13,14^). Although Vrk1^GT3/GT3^ mice have a normal size and lifespan^13^, both males and females are infertile. Vrk1^GT3/GT3^ males display early-onset sterility due to defective proliferation and differentiation of spermatogonia^13,15^, and females are completely sterile due to multiple defects during oocyte development, including defective folliculogenesis^16^, delays in meiotic progression and lagging chromosomes during metaphase II^14^. These defects are consistent with the normally high VRK1 expression in testes^17^. Other than sterility, no symptoms or phenotypes have previously been reported for the Vrk1^GT3/GT3^ mice, even though VRK1 is ubiquitously expressed at moderate levels in most tissues, including in fetal and adult brain and cerebellum^18^.

The complex and severe neuronal phenotypes caused by *VRK1* mutations in humans suggest that VRK1 has essential roles in brain development and in motor neuron survival that have yet to be elucidated. In this study we aimed to determine whether the Vrk1^GT3/GT3^ mouse model has neurological manifestations, and if so, to use these mice to define additional neuronal roles of VRK1. We show that despite decreased expression of *Vrk1* in several neuronal tissues, the neuronal phenotypes in Vrk1^GT3/GT3^ mice are mild, and include a small reduction in brain weight and mild motor impairment. RNA-seq analysis of cortex and spinal cord, comparing samples from Vrk1^+/+^ and Vrk1^GT3/GT3^ mice, yielded several genes with significantly altered expression in the mutant mice. Gene set enrichment analysis (GSEA) of the entire expression data revealed both neuronal and non-neuronal pathways that are affected by *Vrk1* down-regulation and that can be directly related to the phenotypes observed in the mutant mice and in affected individuals with *VRK1* mutations. These results may provide new insights into the pathogenesis of SMA and ALS, the two major forms of motor neuron disease.

## Results

### Vrk1 mRNA expression is reduced in neuronal tissues of Vrk1^GT3/GT3^ mice

**Figure 1:**
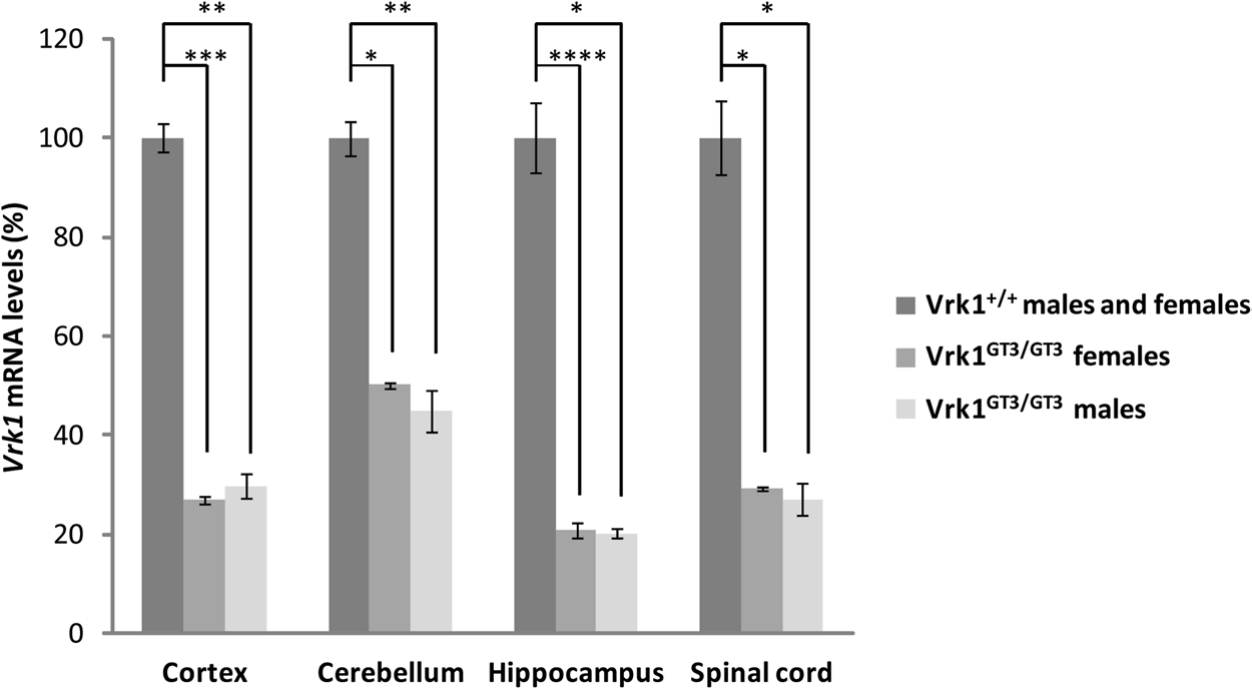
Reduced *Vrk1* expression in neuronal tissues of Vrk1^GT3/GT3^ mutant mice. *Vrk1* mRNA levels in mice Vrk1^GT3/GT3^ vs. Vrk1^+/+^ male and female mice. *Vrk1* mRNA levels were determined by quantitative real-time PCR. mRNA levels of mutant mice are presented as percentage of Vrk1^+/+^ mice levels. Vrk1^+/+^ mRNA levels were calculated for 4 males and 4 females of each genotype (8 mice of each genotype overall). Vrk1^GT3/GT3^ mRNA levels were calculated separately for 4 males of each genotype and 4 females of each genotype. p-values correspond to comparisons between Vrk1^GT3/GT3^ males and females and Vrk1^+/+^ mice of the same sex. *p < 5 × 10^−4^, **p < 5 × 10^−5^, ***p < 5 × 10^−6^, ****p < 5 × 10^−7^. Bars represent means + /− standard deviation. Each experiment was repeated 3 times in duplicates.

Schober *et al*. have previously shown that the *Vrk1* transcript levels are lower in Vrk1^GT3/GT3^ brains relative to Vrk1^+/+^ brains^14^. Using quantitative real-time PCR, we quantified the amount of *Vrk1* mRNA in specific brain regions (cortex, cerebellum and hippocampus) and in the spinal cord of Vrk1^+/+^ and Vrk1^GT3/GT3^ male and female mice. As shown in Fig. 1, both Vrk1^GT3/GT3^ male and female mice had significant, similarly reduced levels, of *Vrk1* mRNA relative to Vrk1^+/+^ mice, in all the tissues examined. *Vrk1* transcript levels ranged from ~20% of control levels in the hippocampus (males: p = 3.3 × 10^−4^, females: p = 5.2 × 10^−7^), 27–30% in the cortex (males: p = 1 × 10^−5^, females: p = 1.6 × 10^−6^), 27–29% in the spinal cord (males: p = 1.5 × 10^−4^, females: p = 5.8 × 10^−5^), and 45–50% in the cerebellum (males: p = 3.5 × 10^−5^, females: p = 7 × 10^−5^).

### Brain mass of Vrk1^GT3/GT3^ mice is significantly smaller, with no reduction in the density of cortical neurons

**Figure 2:**
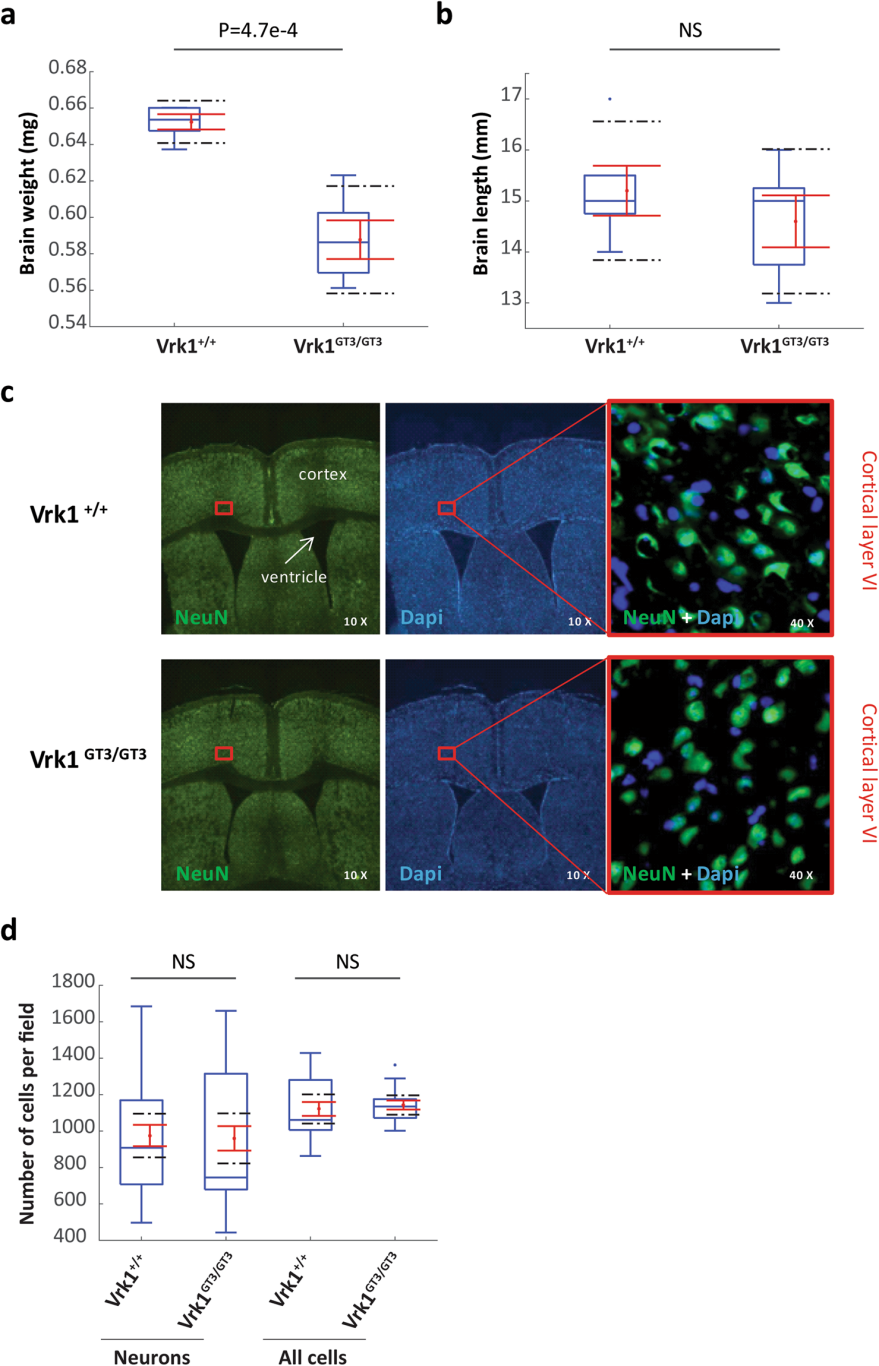
Vrk1^GT3/GT3^ mice brains weigh 10% less than Wild Type brains, with no reduction in the density of cortical neurons). (**a**) The weight and) (**b**) length of Vrk1^+/+^ and Vrk1^GT3/GT3^ brains. n = 5 male brains of each genotype). (**c**) Staining of mouse cortical slices with anti-NeuN antibody (left, 10× magnification, pictures stitched together to show entire slice), DAPI (middle, 10×) and merged (right, 40× magnification). (**d**) Quantitation of number of neurons (NeuN positive) and (**e**) number of total cells (DAPI) in cortical layers IV-VI. n = 5 male brains of each genotype. Cells were counted in 4 slices from each of 5 brains, in fields of equal sizes for each slice. Results are shown as box plots. Boxes (blue) indicate the range between the 25^th^ and 75^th^ percentiles (second and third quartiles), with medians indicated as a horizontal line within the box. Tukey whiskers (vertical lines) extend to the minimal value still within 1.5 interquartile range (IQR) of the lowest quartile and the maximal value still within 1.5 IQR of the upper quartile. Outliers are marked as blue dots. Mean values (dots) and standard errors of the mean (SEM) (bars) are indicated in red. Black dashed lines mark the limits of the 95% confidence interval.

To examine if reduced expression of *Vrk1* recapitulates the congenital microcephaly observed in children with VRK1 deficiency^7–11^, we measured brain weight and length of Vrk1^+/+^ and Vrk1^GT3/GT3^ male mice. We found that Vrk1^GT3/GT3^ brains weighed 10% less (p = 0.00047) than the Vrk1^+/+^ brains (Fig. 2a). Brain length was similar (Fig. 2b). VRK1 is known to play multiple roles in cellular proliferation^1^, and specifically, we have shown that it plays a role in the cell cycle progression of neural progenitors in the developing mouse cortex^8^. To test the hypothesis that the reduced brain mass in Vrk1^GT3/GT3^ mice results from a decrease in the number of cortical neurons, we stained cortex slices with anti-NeuN antibody to identify cortical neurons (Fig. 2c, left) and with DAPI to label all the cells (Fig. 2c, middle). In each mouse, the number of neurons (NeuN positive) and total cells (DAPI) was counted in a field of equal size, representing cortical layers IV-VI. Neither the mean number of neurons, nor the total number of cells per field, differed between Vrk1^+/+^ and mutant mice (Fig. 2d).

### Vrk1^GT3/GT3^ mice show mild motor impairment, with no cognitive deficits

Both cognitive and motor deficits have been described in individuals with bi-allelic *VRK1* mutations^7–12^. In order to examine if reduced *Vrk1* expression causes motor or cognitive impairment, we performed a set of behavioral experiments in Vrk1^GT3/GT3^ vs. Vrk1^+/+^ mice. Motor function was assessed by the rotarod test, (which evaluates motor coordination and balance), and the open field test (assesses general motor function and exploratory activity). Cognitive function was examined using three learning and memory tests: radial arm water maze (spatial learning and memory), novel object recognition (recognition memory) and fear conditioning.

**Figure 3:**
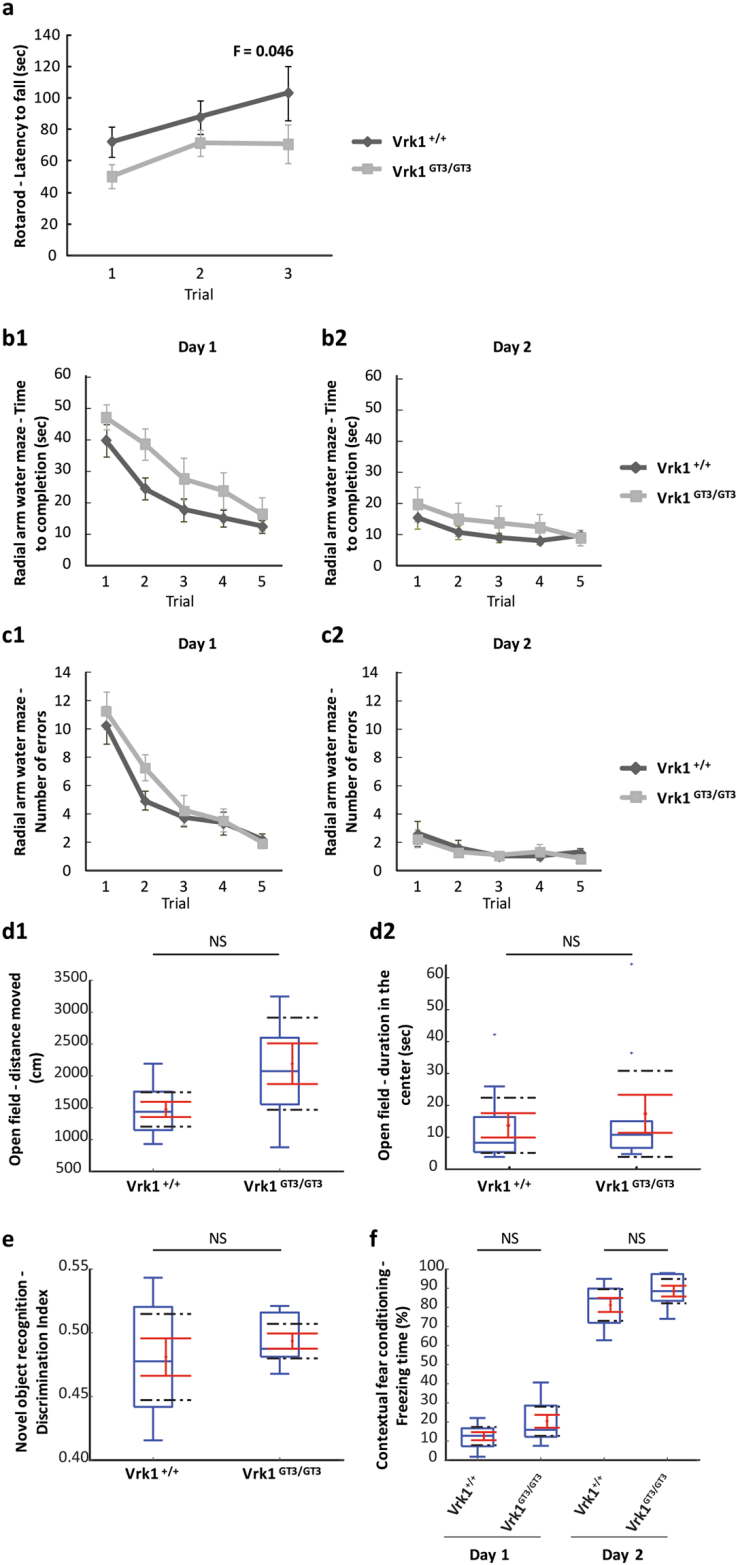
Vrk1^GT3/GT3^ mice show mild motor impairment, with no cognitive deficits. (**a**) Vrk1^GT3/GT3^ mice show impaired motor function in a rotarod test. (**b**–**f**) Vrk1^GT3/GT3^ mice do not show significant motor or cognitive defects in other behavioral tests. (**b**–**c**) Radial arm water maze, measured as the time to completion of the task (reaching the escape platform) for day 1 (**b1**) and day 2 (**b2**); and number of errors for day 1 (**c1**) and day 2 (**c2**). All 5 trials in graphs b and c are mean values of 3 sequential trials in the experiment. (**d**) Open field test. Total distance moved (cm), (**d1**) and duration in the center (sec) (**d2**) are presented. (**e**) Novel object recognition is depicted as a discrimination index defined as the amount of time in which the animal’s head was directed towards the novel object, divided by the sum of times in which the animal’s head was directed to the novel or the familiar object. (**f**) Contextual fear conditioning measured freezing as a percent of total time spent in the experiment device. This is presented for day 1 (habituation, fear conditioning) and day 2 (contextual freezing). n = 10 females of each genotype for all the tests. Graphs (**a**–**c**) show means+/− standard error mean (SEM). Boxplots (**d**–**f**) (blue) indicate the range between the 25^th^ and 75^th^ percentiles (second and third quartiles), with medians indicated as a horizontal line within the box. Tukey whiskers (vertical lines) extend to the minimal value still within 1.5 interquartile range (IQR) of the lowest quartile and the maximal value still within 1.5 IQR of the upper quartile. Outliers are marked as blue dots. Mean values (dots) and standard errors of the mean (SEM) (bars) are indicated in red. Black dashed lines mark the limits of the 95% confidence interval.

The rotarod test showed that the latency to fall for Vrk1^GT3/GT3^ mutant mice was significantly shorter than the Vrk1^+/+^ mice (Fig. 3a, Repeated Measures ANOVA analysis, F = 0.046), showing that these mice have mild motor impairment. In the radial arm water maze, mean time to completion of the task was longer and initial error rates were somewhat higher for the mutant mice, however the differences did not reach significance (Fig. 3b,c). In all the other tests performed (open field, novel object recognition and fear conditioning), no significant differences were observed between Vrk1^GT3/GT3^ mutant and Vrk1^+/+^ mice (Fig. 3d–f).

### RNA-seq analysis of cortex and spinal cord samples reveals that *Vrk1* down-regulation results in differential gene expression

We examined differences in global gene expression to investigate possible mechanisms underlying the phenotypes observed here and in affected individuals with *VRK1* mutations. RNA next generation sequencing (RNA-seq) was performed on total RNA extracted from cortex and spinal cord of Vrk1^+/+^ and Vrk1^GT3/GT3^ mice. RNA-seq confirmed that as observed by real-time PCR (Fig. 1), *Vrk1* expression is reduced in Vrk1^GT3/GT3^ mice, with a 2.9-fold reduction in the cortex (adjusted p-value = 1.18 × 10^−23^) and a 4.6-fold reduction in the spinal cord (adjusted p-value = 1.28 × 10^−31^). Besides *Vrk1*, only a few individual genes showed significant differential expression in the cortex and spinal cord of Vrk1^GT3/GT3^ mice (Tables S1 and S2).

We then looked at expression patterns of gene groups that might contribute to understanding the VRK1 phenotype, and performed a Gene Set Enrichment Analysis (GSEA)^19^ on the entire set of expression data. In this analysis, we examined the Hallmark and KEGG gene set categories of the MSigDB database. In the analysis of the expression data from Vrk1^GT3/GT3^ vs. WT cortices, we found significant enrichment (FDR q-value < 0.01) of gene sets related to cellular proliferation and the DNA damage response, which are known functions of VRK1^1,3,5,6^, confirming the validity of this approach. These pathways include: Mitotic spindle genes (normalized enrichment score [NES] = 3.07, q < 0.00001), Myc targets (NES = −5.8, q < 0.00001), E2F targets (NES = −2.4, q = 0.0008) and DNA repair genes (NES = −3.12, q < 0.00001) (Table 1). Importantly, we also found significant enrichment of neuronal gene sets not previously associated with VRK1 function: neurotrophin signaling (NES = 2.65, q = 0.002) and axon guidance (NES = 2.5, q = 0.004). In addition, we found significant enrichment of gene groups involved in neurodegeneration, including: oxidative phosphorylation genes (NES = −6.06. q < 0.00001) and Proteasome associated genes (NES = −4.02, q < 0.00001).

**Table 1:** Differentially expressed GSEA gene sets enriched in the Vrk1^GT3/GT3^ cortex.

Gene Set Name	Normalized Enrichment score	Number of affected genes	FDR q-value
MITOTIC SPINDLE	3.07	195	<0.00001
NEUROTROPHIN SIGNALING PATHWAY	2.65	118	0.002
AXON GUIDANCE	2.5	124	0.004
E2F TARGETS	−2.4	194	0.0008
DNA REPAIR	−3.12	134	<0.00001
PROTEASOME	−4.2	39	<0.00001
MYC TARGETS V1	−5.8	187	<0.00001
OXYDATIVE PHOSPHORYLATION	−6.06	106	<0.00001

## Discussion

In this work, we investigated both behavioral features and neuronal gene expression in mice with a partial knockout of *Vrk1*. In humans, VRK1 related disease is recessive, and as we have previously shown, R358X is subject to nonsense mediated decay and results in lack of VRK1 protein^8^. Although other disease-associated mutations have not been fully characterized, the phenotypes they cause are similar to those caused by the R358X mutation, and since inheritance is recessive in all cases, they are also likely to be loss-of-function mutations^7,9–12^. Vrk1^GT3/GT3^ mice, which express low amounts of *Vrk1* due to a gene trap insertion, could be a suitable model to study neuronal functions of VRK1. *Vrk1* mRNA levels in neuronal tissues of Vrk1^GT3/GT3^ males and females were found to be lower than in Vrk1^+/+^ mice, ranging from ~20% in the hippocampus to ~50% in the cerebellum (Fig. 1), similar to previous reports in other organs^13,14^. Vrk1^GT3/GT3^ mice had significantly smaller brains (10% lighter, p = 0.00047) than Vrk1^+/+^ mice (Fig. 2a), consistent with the microcephaly observed in children with *VRK1* mutations. Evaluation of motor and cognitive abilities of Vrk1^GT3/GT3^ mice revealed mild motor impairments in the rotarod test (Fig. 3a). In all the other experiments: Radial arm water maze, fear conditioning, open field and novel object recognition, no significant differences were observed between mutant and VRK1^+/+^ mice (Fig. 3b–f).

Vrk1^GT3/GT3^ mice show a mild neuronal phenotype, However they do not recapitulate the severe neuronal phenotype seen in humans with *VRK1* mutations. While human neurological phenotypes are often not reproducible in mice models^20^, the most likely explanation of the milder phenotype in this case is the substantial residual level of functional *Vrk1* mRNA (Fig. 1)^13,14^, as opposed to the lack of protein, as observed in R358X human patients^8^. While reduced expression of *Vrk1* causes sterility due to defects in gamete production in male and female mice^13–16^, reflecting the need for high Vrk1 expression in gonadal tissue^17^, our results show that reduced *Vrk1* expression is sufficient to result in only minor neurological impairment. Redundancy of the VRK proteins may also contribute to the milder phenotype of *Vrk1* knockdown in mice: mammals have 2 additional members of the VRK family members, VRK2 and VRK3^18,21^, and some functions of the VRK proteins overlap, e.g. p53 phosphorylation and promotion of cell cycle progression^22–26^. In humans, this redundancy is not protective for the neurological manifestations in VRK1-null individuals, but in the Vrk1^GT3/GT3^ mouse, the nervous system may be less sensitive than the reproductive system and this redundancy may compensate for the partial deficiency of Vrk1. Nevertheless, moderate reductions (50–75%) in *Vrk1* expression in the nervous system were still associated with reduced brain mass and mild motor impairment.

To examine the effect of *Vrk1* down-regulation in the neuronal tissues affected in humans with *VRK1* mutations, and to reveal pathways that may underlie the neuronal phenotypes of both the Vrk1^GT3/GT3^ mice and affected humans, we performed transcriptome analysis using RNA-seq from RNA derived from the cortex and spinal cord of Vrk1^GT3/GT3^ and Vrk1^+/+^ mice. In agreement with our real-time PCR results, *Vrk1* expression was reduced in both tissues, and this was correlated with highly significant changes in gene expression for only a number of genes (Tables S1 and S2). In order to assess transcriptomic changes caused by the partial *Vrk1* knockout, we performed GSEA analysis, which determines whether an *a priori* defined set of genes shows statistically significant, concordant differences between two biological states. Rather than assessing individual genes, GSEA analyzes groups of genes belonging to the same pathways. This was done in order to identify gene expression patterns that reflect processes or pathways affected by *Vrk1* down-regulation. The validity of this analysis was confirmed by the identification of gene sets for processes in which VRK1 is known to be involved, e.g. those related to cellular proliferation (mitotic spindle, Myc targets, E2F targets) and the DNA damage response. The proliferation-related gene sets enriched in the Vrk1^GT3/GT3^ cortex may be directly related to the smaller brains of the Vrk1^GT3/GT3^ mice and to the microcephaly phenotype observed in *VRK1* homozygous affected humans. The smaller brain mass of Vrk1^GT3/GT3^ mice was not associated with reduced density of cortical neurons, and may be the result of reduced non-neuronal mass and/or reduction in the total number of cortical neurons. We have previously demonstrated that *in-utero* knockdown of Vrk1 reduces proliferation of neural precursors in the developing brain^8^. Expression analysis in this study indicated impairment of a number of proliferation-related pathways that may underlie reduced brain mass. Significantly affected pathways included mitotic spindle genes, which are implicated in primary microcephaly^27,28^, Myc signaling, which increases proliferation and self renewal of neural progenitor cells^29,30^ and E2F targets, that play a role in proliferation^31^, migration^32^ and apoptosis^33^ of neurons in the developing central nervous system.

Beyond proliferation-related gene sets, we also observed down-regulation of DNA repair pathways. This is consistent with the known roles of VRK1 in the DNA damage response (specifically the response to DNA double strand breaks^3,5,6,24^), placing VRK1 as one of several DDR genes in which mutations cause microcephaly^27,28^ and/or neurodegeneration^34,35^. To summarize, morphological and gene expression analysis of Vrk1^GT3/GT3^ mice highlight the importance of VRK1 in neuronal proliferation and the DNA damage response, roles that are important for brain development.

In addition to functions in which VRK1 is known to play a role, GSEA analysis revealed neurotrophin signaling and axon guidance as two novel neuronal pathways affected by Vrk1 deficiency. Neurotrophins play multiple roles in the developing and adult nervous system. During embryonic development, neurotrophins, through binding to their receptors, induce differentiation of neural progenitors, regulate survival and apoptosis of neurons, and play roles in axon growth and dendrite growth and pruning. In the adult brain, neurotrophins control synaptic function and plasticity, and sustain neuronal survival, morphology and differentiation^36^. There is growing evidence that neurotrophins have a neuroprotective role, but they are also capable of inducing neuronal apoptosis^37,38^. Loss of survival-promoting neurotrophic signaling has been proposed as a contributing factor to neurodegenerative disorders including ALS^37–39^. However, increased expression levels of several neurotrophic factors were reported in several cases of ALS and were thought to play a defensive role against existing pathology^37,38,40–45^. Our GSEA analysis revealed that in the cortex of Vrk1^GT3/GT3^ mice, neurotrophin signaling genes were up-regulated (Table 1). Interestingly, neurotrophin signaling was also among the pathways enriched in an expression array analysis of RNA derived from WT human B cells versus RNA from a VRK1 human patient^8,46^. Together, our results suggest that neurotrophin signaling may be affected by VRK1 deficiency, either because VRK1 is part of the neurotrophin pathway, or as a secondary effect, perhaps as a compensatory mechanism aimed at coping with *VRK1* down-regulation.

Axon guidance proteins, found to be up-regulated in the cortex of Vrk1^GT3/GT3^ mice, also have well established roles in neurodevelopment, and are also implicated in the pathogenesis of ALS^47^. During development, axon guidance proteins can attract or repel axons, guiding them to their synaptic target sites. In the adult nervous system, axon guidance proteins are important for the function of synapses, including the neuromuscular junction (NMJ), which is the focal point of both SMA and ALS pathology. Altered expression of axon guidance genes has been observed in neurons and muscles of ALS patients and mouse models of ALS, and may initiate or facilitate NMJ changes in the pre-symptomatic stages of ALS^47–49^. Remarkably, we also observed changes in axon guidance genes in fibroblasts and neural precursors derived from a patient homozygous for the *VRK1*-R358X mutation (Manuscript in preparation). Our results suggest a possible involvement of axon guidance genes in the neuronal pathology caused by *VRK1* mutations. Further research is needed to confirm such an involvement.

Two other gene sets identified in our analysis were proteasome-related genes and oxidative phosphorylation. While these pathways are not specific to the nervous system they have been linked to the pathogenesis of ALS^50,51^. The ubiquitin-proteasome system is responsible for protein degradation, and failure to degrade mis-folded proteins results in their aggregation in cellular inclusions, which is a hallmark of ALS^38,51^. Proteasome related gene expression changes were observed in multiple transcriptome studies using neuronal samples from ALS patients and animal models^52,53^. Another feature of ALS is mitochondrial dysfunction, which is one of the earliest pathophysiological events in the disease. Disruption of mitochondrial structure, dynamics, bioenergetics and calcium buffering has been extensively reported in ALS patients and model systems^54^. In this study, we observed down-regulation of oxidative phosphorylation genes, which is consistent with impaired expression of genes related to mitochondrial function^52,53,55^.

Our expression studies from the Vrk1^GT3/GT3^ mice thus link VRK1 to mechanisms known to be affected in other, much more common cases of ALS. VRK1-associated disease in humans includes neurodegeneration in all cases and has been classified as SMA or early-onset ALS. These manifestations occur after years of accumulating damage; the lifespan of mice may not be long enough for such deficits to be revealed^56^. The combination of the human VRK1 phenotype with the mild motor neuron symptoms presenting in Vrk1^GT3/GT3^ mice along with the gene expression changes observed, suggest that modeling a full-fledged neuronal knockout of Vrk1 in the mouse may recapitulate the human disorder.

In summary, we showed that mice expressing reduced levels of *Vrk1* (20–50% of WT levels) have smaller (10%) brain size and suffer mild neurological impairment. The presence of a neuronal phenotype despite substantial residual expression of Vrk1 suggests that developing a mouse completely lacking Vrk1 in the nervous system would facilitate further analysis of VRK1’s neuronal roles. Analysis of gene expression data from Vrk1^GT3/GT3^ mutant cortices highlighted previously known functions of VRK1 in cellular proliferation and the DNA damage response, which are both relevant to microcephaly. Importantly, they revealed novel neuronal pathways in which VRK1 may be involved, including neurotrophin signaling and axon guidance, as well as general processes implicated in the pathogenesis of ALS, e.g. the proteasome and oxidative phosphrylation. Our results, combined with the SMA/ALS phenotype observed in humans with VRK1 deficiency, suggest the pathology caused by *VRK1* mutations may share common mechanisms with, both SMA and ALS. These neuropathies have previously been considered to be separate entities, and only recently has evidence emerged to suggest that shared mechanisms underlie their pathogenesis^54,56–59^. Our work provides a basis for future studies of the neuronal functions of VRK1, which may impact other, more common forms of motor neuron disease. In addition, the observation of minimal neurological impairment in the partial knockout mouse may be clinically significant, since it suggests that restoration of even small amounts of VRK1 activity in *VRK1* mutant patients could be of therapeutic value.

## Materials and Methods

### Animals

Heterozygous Vrk1^GT3/+^ mice were kindly provided by Prof. Paula Traktman from the Medical University of South Carolina, and bred to obtain homozygote mutant (Vrk1^GT3/GT3^) and WT (Vrk1^+/+^) mice. All procedures were performed according to the guidelines of the Institutional Animal Care and Use Committee (IACUC) of the Hebrew University, which is an AAALAC internationally accredited institute. Animal protocols were approved by the Hebrew University IACUC. For behavioral tests, 10 Vrk1^+/+^ and 10 Vrk1^GT3/GT3^ 6 month old females were used. For mRNA expression analysis, total RNA was extracted from brains and spinal cords of 4 Vrk1^+/+^ males, 4 Vrk1^+/+^ females, 4 mutant males and 4 mutant females (6 month old). For brain size measurements and cortical neuron count, we used 5 Vrk1^+/+^ and 5 Vrk1^GT3/GT3^ 6 month old males. RNA-Seq was performed on spinal cord samples from 3 Vrk1^+/+^ and 3 Vrk1^GT3/GT3^ 6 month old females and cortex samples from 4 Vrk1^+/+^ and 3 Vrk1^GT3/GT3^ 6 month old females.

### Quantitative Real-Time PCR

Tissues were disrupted and total RNA extracted using Tri-Reagent. RNA was reverse transcribed with ImProm-II reverse transcriptase (Promega) using random hexamers in the presence of RNase inhibitor (RNasin, Promega). Quantitative Real Time PCR reactions were performed using Power SYBR master mix (Applied Biosystems). Ct values were normalized to the Ct values of the mouse housekeeping gene Tbp. Relative mRNA levels were calculated as 2^−ΔCt^. Each experiment was repeated 3 times with duplicates. For *Vrk1* mRNA expression, we used primers located downstream from the GT3 insertion.

### Immunohistochemistry and cell count

Brains were fixed in 4% paraformaldehyde and frozen in Tissue Tek OCT compound. Cryostat sections were cut coronally at 10 microns and immunostained with mouse anti- NeuN antibody (Millipore, MAB377) together with alexafluor 488 conjugated goat anti mouse (Life Technology, A11001). Nuclei were stained with DAPI. Photos were taken with a fluorescent microscope and NeuN-positive cells and DAPI-stained nuclei were counted separately in a field of equal size for each slice.

### Behavioral tests

Apart from the rotarod and fear conditioning tests, all behavioral experiments were monitored by a digital video camera connected to a computer using EthoVision XT 11 tracking software.

### Rotarod

Mice were placed on a rotarod apparatus with an initial rotating speed of 5 revolutions per minute. The speed of rotation was escalated in a fixed cline to a maximal speed of 40 rpm after 99 seconds. Latency of each animal to fall from the device was recorded, with a cut off of four minutes before the trial was terminated had the animal not fallen from the device. 3 sequential trials were performed, with 20 minute intervals between trials.

### Open field

Each animal was placed in the corner of a 50 × 50 × 30 cm (height) white plastic arena. The animal was then left to freely move in the arena for the duration of 6 minutes test time. Total distance moved, time spent between the arena’s border zone (peripheral 10 cm on each size) and center (remainder of the arena) and the animal transitions between these zones were monitored.

### Novel object recognition

The novel object recognition test was composed of 2 stages: (A) Habituation stage: each animal was placed in a 25 × 25 white plastic compartment, where two similar plastic objects (about 3 × 3 × 2 cm (height) each) were glued to the arena floor. The animal was then left to freely investigate the arena and the objects for 10 minutes. After that, the animal was returned to its home cage for 1 hour. (B) Test stage: After 1 hour elapsed, the animal was returned to the same compartment it was in during the habituation phase, only now the arena contained one object identical to those used in the habituation phase (familiar object) and another object, different in its shape, size, color and texture (novel object). During the 4 minute period, the time spent with its head directed to each object was recorded. To evaluate novel object preference, we used the discrimination index (measurement of the attention given the novel object divided by the sum of the attentions given to both objects) for duration (in sec) of head directed towards objects.

### Contextual fear conditioning

The fear conditioning test was conducted inside an apparatus (Panlab) built of a double box (external part and an internal one to which an animal is introduced). The device has a grid floor capable of transmitting low electrical current in short time intervals and is operated by computer using Packwin software. The protocol is composed of 2 stages: (A) Fear conditioning: on the first day of the protocol (training) every animal was introduced to the chamber, a tone was sounded for 20 seconds followed by a 0.5 milliampere electric shock administered via the grid floor. Afterwards the tone was a repeated for another 20 seconds and followed by the same foot shock. Subsequently, the animal was returned to its home cage. (B) Contextual freezing: On the second day, each animal was again put in the same chamber in which it was exposed to the conditioning protocol the previous day but without auditory signals, and the fraction of time spent freezing was recorded over a 5 minute test time.

### Radial arm water maze

The radial arm water maze (RAWM) contains six swim paths (arms) extending out of an open central area, with an escape platform located at the end of one arm (the goal arm). The goal arm location remains constant for a given mouse, while the start arm (the arm in which the mouse is placed in each trial) changes from trial to trial. On day 1, mice are trained for 15 trials (spaced over 3 h), with trials alternating between visible and hidden platforms. On day 2, mice perform 15 trials with the hidden platform. Entry into an incorrect arm is scored as an error. The number of errors and the amount of time required for each mouse to find the escape platform were recorded. The exact protocol was performed as described previously^60^. The average value of every 3 trials for each animal (errors and time to completion) was calculated and used for statistical analysis.

### Library preparation and RNA-seq

Quality of RNA extraction, yield and Library synthesis were measured using RNA ScreenTape kit (AGILENT TECHNOLOGIES), D1000 ScreenTape kit (AGILENT TECHNOLOGIES), Qubit® RNA HS Assay kit (Invitrogen), Qubit® DNA HS Assay kit (Invitrogen) were used for each specific step purpose. For mRNA library preparation, KAPA Stranded mRNA-Seq Kit with mRNA Capture Beads (kappa biosystems, KK8421, https://www.kapabiosystems.com/) was used. In brief,  1 μg of total RNA was used for the library construction, libraries were eluted in 20 μl of elution buffer, and adjusted to 10 nM. 10 μl (50%) from each sample were collected and pooled in one tube. Multiplex samples were pooled (1.5 pM including PhiX 1.5%) and loaded in a NextSeq 500/550 High Output v2 kit (75 cycles) cartridge (Illumina) and loaded on a NextSeq 500 System (Illumina), with 75 cycles and single-Read Sequencing conditions.

### RNA-seq data analysis

Raw reads (fastq files) were quality-trimmed at both ends, adapter sequences were removed with cutadapt (version 1.11, http://cutadapt.readthedocs.org/en/stable/), filtering out reads shorter than 15 nt. Reads were further filtered to remove very low quality reads, using the fastq_quality_filter program of the FASTX package (version 0.0.14, http://hannonlab.cshl.edu/fastx_toolkit/), with a quality threshold of 20 at 90 percent or more of the read’s positions. The processed fastq files were mapped to the mouse transcriptome and genome using TopHat (v2.0.14). The genome version was GRCm38, with annotations from Ensembl release 84. Mapping allowed up to 5 mismatches per read, a maximum gap of 5 bases, and a total edit distance of 10. Quantification was done using htseq-count (version 0.6.0, http://www-huber.embl.de/users/anders/HTSeq/doc/count.html). Strand information was set to ‘reverse’, and an annotation file lacking information for genes of type IG, TR, Mt, rRNA, tRNA, miRNA, misc_RNA, scRNA, snRNA, snoRNA, sRNA, scaRNA, piRNA, vaultRNA, ribozyme, artifact and LRG_gene, was used. Normalization and differential expression were assessed with the DESeq2 package (version 1.12.4). Genes with a sum of counts less than 10 over all samples were filtered out prior to normalization. Differential expression was calculated mutant and wild type samples within each tissue (cortex or spinal cord). The significance threshold for all comparisons was taken as padj <0.1 (default).

### Gene Set Enrichment analysis (GSEA)

Whole expression data was subjected to gene set enrichment analysis using GSEA^19^ with the corresponding human ortholog gene symbols. Human orthologs were extracted from Ensembl^61^. GSEA uses complete expression data (cut-off independent) to determine whether a-priori defined sets of genes show statistically significant, concordant differences between two biological states. Gene sets of the MSigDB database categories H and KEGG were examined (MsigDB v6.1, May 2017 release). The significance threshold was FDR q value < 0.01.

### Statistical analysis

For the rotarod, open field, radial arm water maze and fear conditioning tests, results were analyzed using repeated measures ANOVA. The results of the novel object recognition tests were analyzed using student’s *t*-test. Statistical analysis for mRNA expression levels (quantitative real-time PCR), brain length, brain weight and cortical neurons/total cell counts were performed using paired, 2 tailed *t*-test with p < 0.05 considered significant.

### Data availability statement

The RNA-seq data will be deposited to NCBI’s Gene Expression Omnibus (GEO).

## Electronic supplementary material

Supplementary data
