# Supplementary material for: Vrk1 partial Knockdown in Mice Results in Reduced Brain Weight and Mild Motor Dysfunction, and Indicates Neuronal VRK1 Target Pathways
Source: Sci Rep. 2018 Jul 26;8:11265. doi: 10.1038/s41598-018-29215-x (PMC6062608; doi:10.1038/s41598-018-29215-x)
Supplement: Supplementary file 1 — Supplementary data [file 41598_2018_29215_MOESM1_ESM.pdf]

Vrk1 partial Knockdown in Mice Results in Reduced Brain Weight and Mild Motor Dysfunction, and Indicates Neuronal VRK1 Target Pathways

Hadar Vinograd-Byk, Paul Renbaum and Ephrat Levy-Lahad

Supplementary data

Table S1: Genes showing significantly altered expression in the cortex of Vrk1$^{GT3/GT3}$ mice

Gene	Description	Fold change	p value	Padj (Benjamini-Hochberg)
Vrk1	Vaccinia related kinase 1	-2.89	1.24E-27	1.18E-23
Xaf1	XIAP associated factor 1	2.69	1.58E-08	5.02E-05
Tekt1	Tektin1	3.52	7.59E-08	1.81E-04
Serpina3n	Serine (or cysteine) peptidase inhibitor, clade A, member 3N	8.79	1.12E-34	2.14E-30
Serpina3m	Serine (or cysteine) peptidase inhibitor, clade A, member 3M	236.71	3.86E-13	1.84E-09

Table S2: Genes showing significantly altered expression in the spinal cord of Vrk1$^{GT3/GT3}$ mice

Gene	Description	Fold change	p value	Padj (Benjamini-Hochberg)
Vrk1	Vaccinia related kinase 1	-4.62	6.253E-36	1.27E-31
Cox6a2	Cytochrome c oxidase subunit Via polypeptide 2	3.43	2.260E-05	4.19E-02
Serpina3n	Serine (or cysteine) peptidase inhibitor, clade A, member 3N	3.59	8.521E-12	4.34E-08
Xaf1	XIAP associated factor 1	5.2	4.872E-19	3.3E-15
Foxg1	Forkhead box G1	8.05	1.696E-05	3.46E-02
Tbr1	T-box brain gene 1	23.97	6.800E-06	1.54E-02
Serpina3m	Serine (or cysteine) peptidase inhibitor, clade A, member 3M	187.4	7.012E-10	2.86E-06
